# Supplementary material for: Endonucleolytic RNA cleavage drives changes in gene expression during the innate immune response
Source: bioRxiv. 2023 Oct 1:2023.09.01.555507. Originally published 2023 Sep 2. Preprint. [Version 2] doi: 10.1101/2023.09.01.555507 (PMC10491309; doi:10.1101/2023.09.01.555507)

bioRxiv preprint doi: https://doi.org/10.1101/2023.09.01.555507; this version posted October 1, 2023. The copyright holder for this preprint (which was not certified by peer review) is the author/funder. This article is a US Government work. It is not subject to copyright under 17 USC 105 and is also made available for use under a CC0 license.

S1 Related to Figure 1. A Cleavage patterns of total RNA extracts show activation of RNase L in 2-5A or poly I:C treated WT, but not *RNASEL* KO, A549 cells. rRNA cleavage assay performed on a BioAnalyzer. Black arrows indicate the 28 and 18S rRNAs and red arrows show RNase L degradation products. **B** Heatmap representation of the most highly expressed genes during RNase L activation. Trends are consistent across replicates. **C** Western blots show that transcription factors FOSB and JUN do not increase in *RNASEL* KO cell due to 2-5A activation. **D** Western blots show JNK, p38 and ZAKα

bioRxiv preprint doi: https://doi.org/10.1101/2023.09.01.555507; this version posted October 1, 2023. The copyright holder for this preprint (which was not certified by peer review) is the author/funder. This article is a US Government work. It is not subject to copyright under 17 USC 105 and is also made available for use under a CC0 license.

are not activated in *RNASEL* KO cells after 2-5A treatment. **E** Western blots show ERK is not activated in WT cells after 2-5A treatment.

S2 Related to Figure 2. Western blots show no detectable effect of RNase L activation on NF-κB activation by poly I:C and no detectable activation by 2-5A. ERK activation was not observed during 2-5A or poly I:C treatment.

S3 Related to Figure 3. A Cleavage of RNAs in the cell occurs when RNase A is electroporated into cells, but not when BSA is electroporated, as observed by rRNA cleavage assays performed on BioAnalyzer. Arrows indicate the 28S and 18S rRNAs. **B** Volcano plot showing upregulation of example proinflammatory cytokines and transcription factors during RNase A electroporation. Differentially expressed genes define as $p_{adjusted}$ value <0.05, log_2fold change >1.

S4 Related to Figure 4. A Increased 5'UTR:ORF ratios indicating higher relative uORF translation when active RNase is present in the cell, but not in controls (-2-5A and BSA electroporated). **B** Normalized average ribosome footprint occupancy (metagene plot) around the start codon of main ORFs reveals increased relative ribosome footprint levels in the 5' UTRs when an active RNase is present vs the respective control.
In all panels data shown for RNase L activation (+2-5A) was obtained from [21].

S5 Related to Figure 6. A Ribosome profiling tracks for gene model of *ATF4* and *CHOP* in WT and *RNASEL* KO cells during 2-5A or poly I:C treatment. Data show the poly I:C dependent shift toward main ORF vs 5'UTR translation is greater when RNase L is absent. Asterisks show RNase L dependent ribosome profiling peaks in 2-5A and poly I:C treated cells that likely correspond to altORF translation initiation events. **B** 5'UTR:main ORF ratios computed from ribosome profiling data in WT and *RNASEL* KO cells during poly I:C treatment for *ATF4* and *CHOP*.

S6 Related to Figure 7. A uORFs were identified on *IFIT2* and *3* mRNAs based on the increase in ribosome densities on alternative start codons (CUG or GUG, green markings)

bioRxiv preprint doi: https://doi.org/10.1101/2023.09.01.555507; this version posted October 1, 2023. The copyright holder for this preprint (which was not certified by peer review) is the author/funder. This article is a US Government work. It is not subject to copyright under 17 USC 105 and is also made available for use under a CC0 license.

in poly I:C treated WT or *RNASEL* KO cells. Red marking shows the start codon for the main ORF. **B** Western blot for *IFIT1* and *IFIT2* shows increased protein levels in poly I:C treated RNase L KO cells, suggesting control of TE or RNA degradation reduce levels in WT cells.

Table S1. Results tables from DESeq2 analysis for RNA-seq (WT and *RNASEL* KO, +/- 2-5A, +/- poly I:C, BSA vs RNase A electroporated).

Table S2. Results tables from DESeq2 analysis for ribosome profiling (WT and *RNASEL* KO, +/-2-5A, +/- poly I:C).

Table S3. List of genes that are upregulated by JNK/p38 in WT 2-5A treated cells and interferon response in WT poly I:C treated cells. This list was used to create violin plots in Figures 2D and 3D. In addition, a longer list of all genes related to JNK/p38 or interferon are given, as derived from the Harmonizome and Hallmark datasets, respectively (see Methods), used in Figures 2C and 3C.

bioRxiv preprint doi: https://doi.org/10.1101/2023.09.01.555507; this version posted October 1, 2023. The copyright holder for this preprint (which was not certified by peer review) is the author/funder. This article is a US Government work. It is not subject to copyright under 17 USC 105 and is also made available for use under a CC0 license.

Figure S1

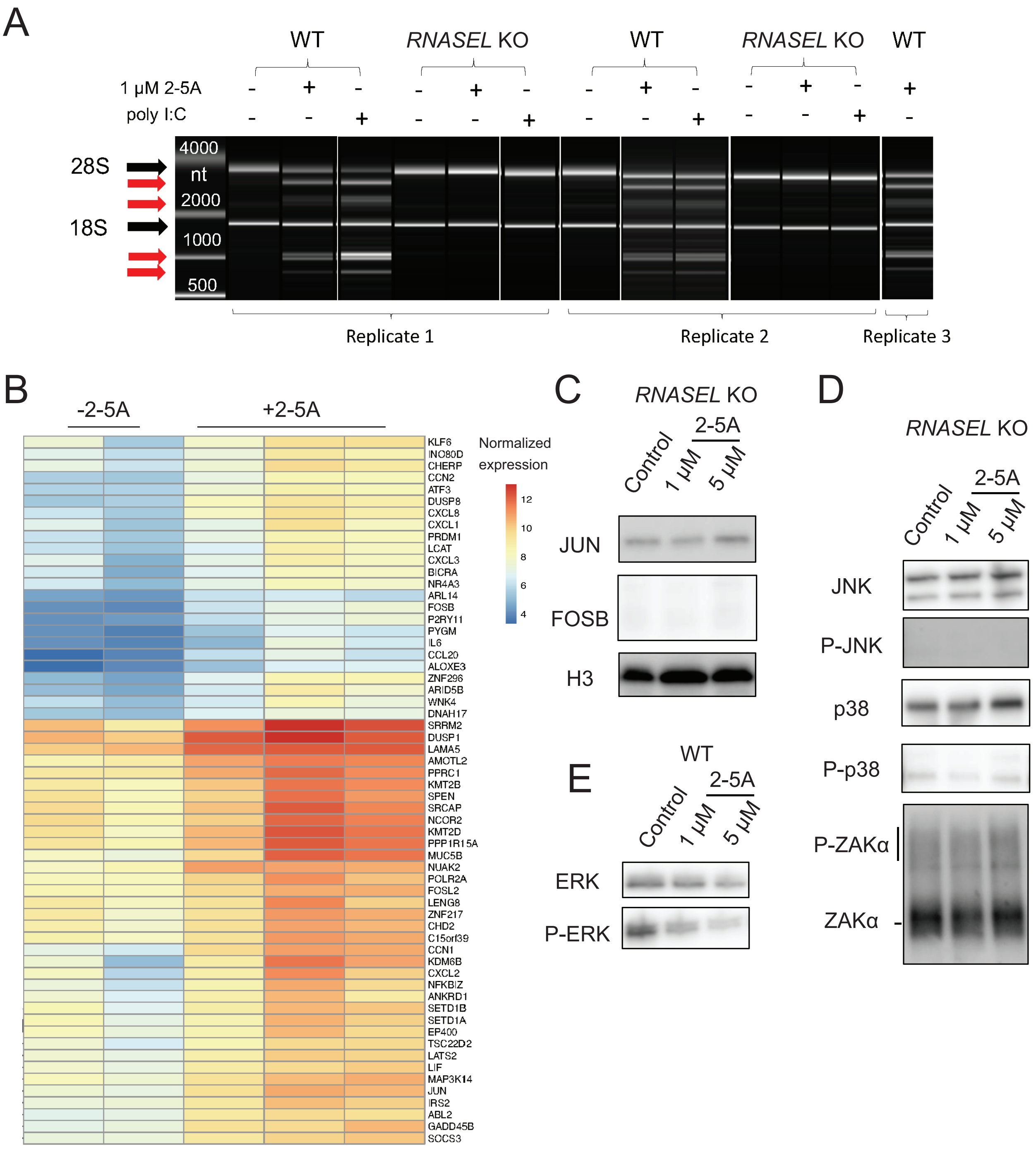

bioRxiv preprint doi: https://doi.org/10.1101/2023.09.01.555507; this version posted October 1, 2023. The copyright holder for this preprint (which was not certified by peer review) is the author/funder. This article is a US Government work. It is not subject to copyright under 17 USC 105 and is also made available for use under a CC0 license.

Figure S2

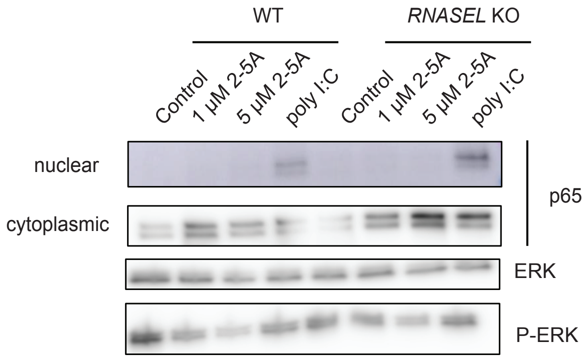

bioRxiv preprint doi: https://doi.org/10.1101/2023.09.01.555507; this version posted October 1, 2023. The copyright holder for this preprint (which was not certified by peer review) is the author/funder. This article is a US Government work. It is not subject to copyright under 17 USC 105 and is also made available for use under a CC0 license.

Figure S3

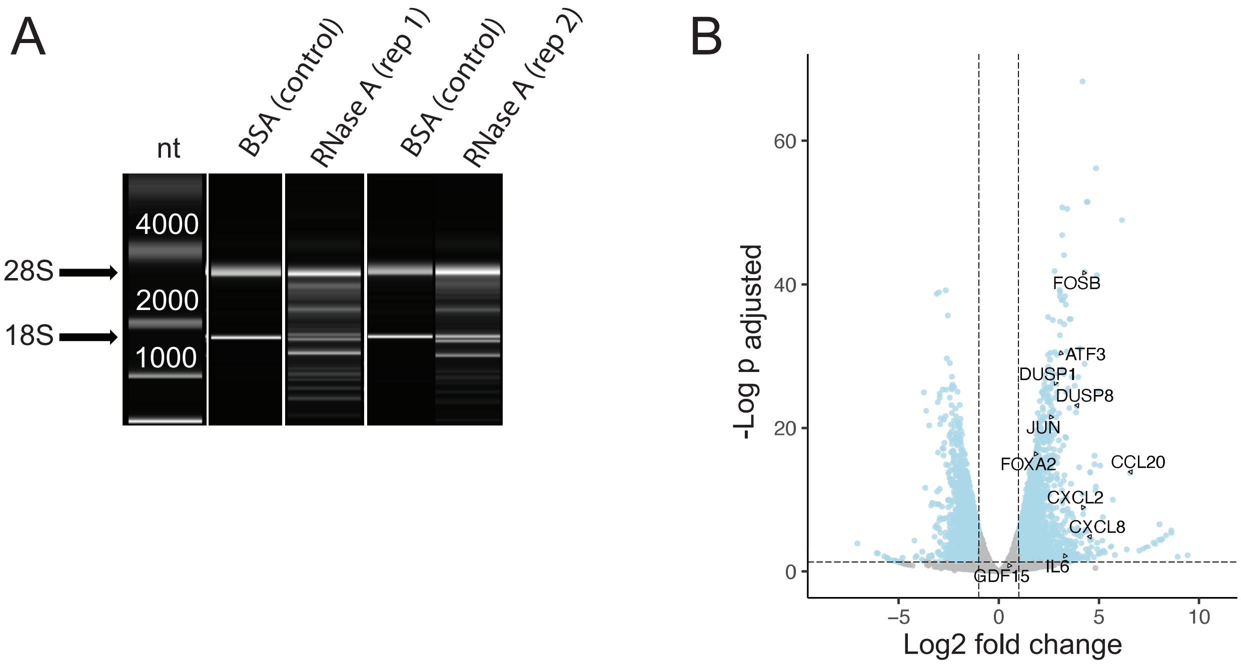

bioRxiv preprint doi: https://doi.org/10.1101/2023.09.01.555507; this version posted October 1, 2023. The copyright holder for this preprint (which was not certified by peer review) is the author/funder. This article is a US Government work. It is not subject to copyright under 17 USC 105 and is also made available for use under a CC0 license.

Figure S4

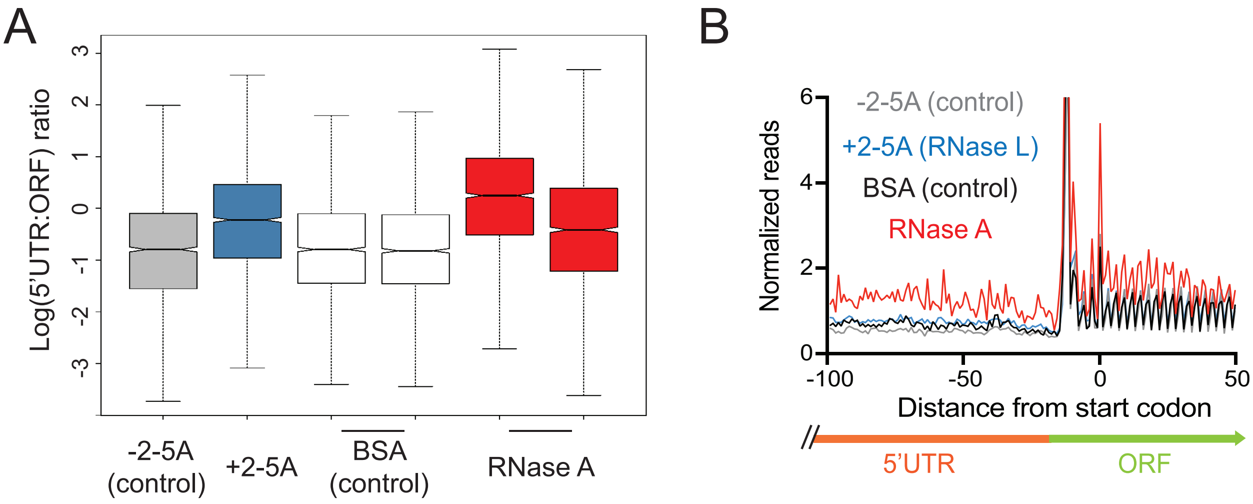

bioRxiv preprint doi: https://doi.org/10.1101/2023.09.01.555507; this version posted October 1, 2023. The copyright holder for this preprint (which was not certified by peer review) is the author/funder. This article is a US Government work. It is not subject to copyright under 17 USC 105 and is also made available for use under a CC0 license.

Figure S5

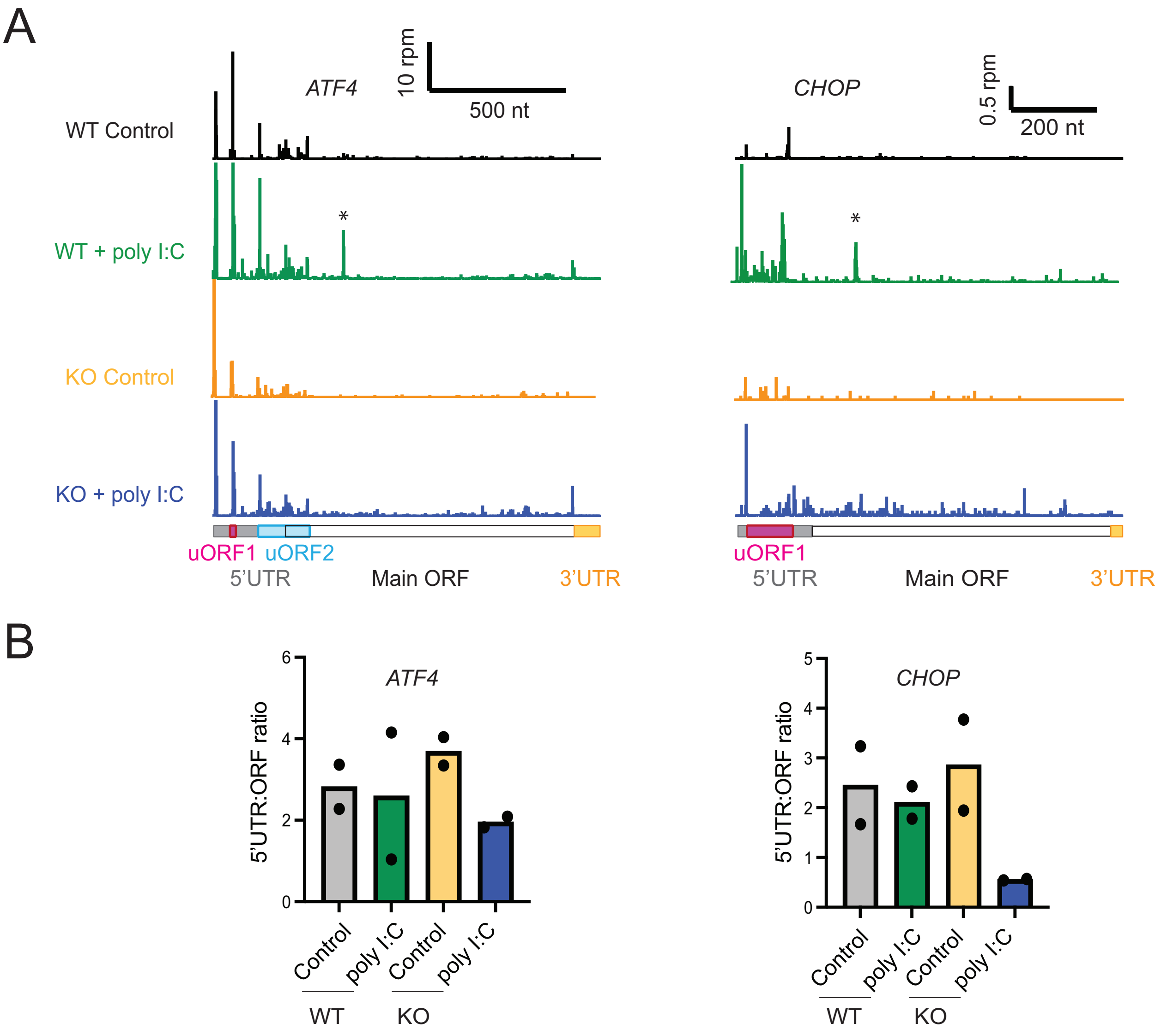

bioRxiv preprint doi: https://doi.org/10.1101/2023.09.01.555507; this version posted October 1, 2023. The copyright holder for this preprint (which was not certified by peer review) is the author/funder. This article is a US Government work. It is not subject to copyright under 17 USC 105 and is also made available for use under a CC0 license.

Figure S6

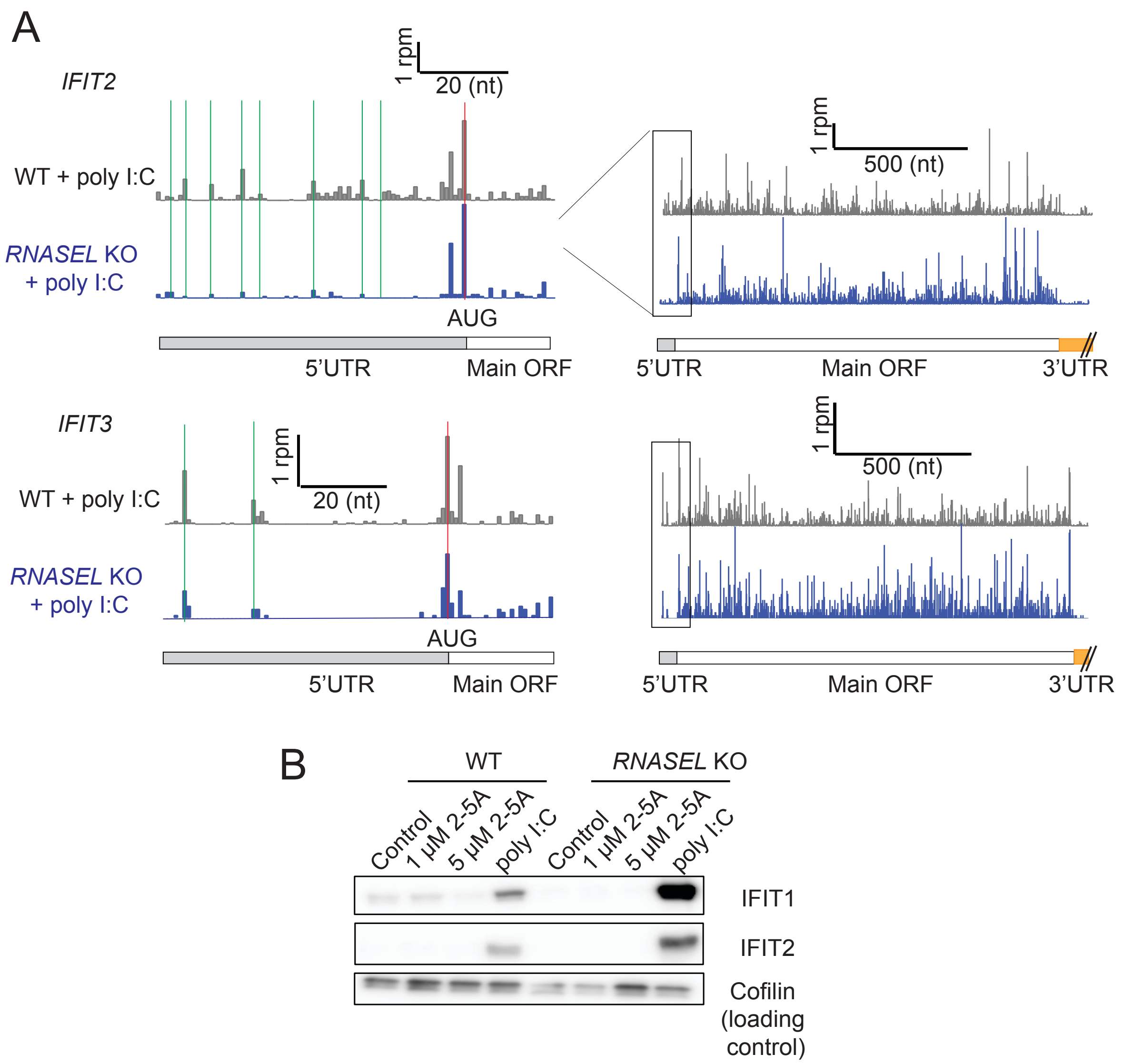

Supplement: Supplement 4 [file NIHPP2023.09.01.555507v2-supplement-4.pdf]
